# The effect of atmospheric pressure on oxygen saturation and dyspnea: the Tromsø study

**DOI:** 10.1007/s00484-020-01883-3

**Published:** 2020-03-03

**Authors:** Lisa M. E. Dohmen, Mark Spigt, Hasse Melbye

**Affiliations:** 1 Department of Family Medicine, CAPHRI, Maastricht University, P.O. Box 616, 6200 MD Maastricht, The Netherlands; 2 General Practice Research Unit, Department of Community Medicine, UiT The Arctic University of Norway, Tromsø, Norway

**Keywords:** Atmospheric pressure, Oxygen saturation, Dyspnea, Sea level, Daily pressure changes

## Abstract

A drop in atmospheric pressure, as observed at high altitudes, leads to decreased oxygen saturation. The effect of regular changes in barometric pressure at sea level has never been studied in a general population. A cohort of adults aged 40 years were examined with pulse oximetry at two separate visits, and the local barometric pressure was available from the local weather station. The study aimed at determining the effect of atmospheric pressure on oxygen saturation also called SpO_2_, as well as on shortness of breath. Based on spirometry, the participants were divided into two groups, with normal and decreased lung function. Decreased lung function was defined as forced expiratory volume in 1 s (FEV_1_) below lower limit or normal (LLN) or FEV_1_/FVC (FVC, forced vital capacity) below LLN, with GLI 2012 reference values. The statistical analysis included uni/multivariable linear and logistic regression. A total of 7439 participants of the Tromsø 7 cohort study were included. There was a significant association between barometric pressure and SpO_2_ < 96%, and we found that a reduction of 166.67 hPa was needed to get a 1% reduction in SpO_2_. The change in atmospheric pressure was not significantly associated with shortness of breath, also not in subjects with reduced lung function.

## Introduction

The respiratory system is influenced by weather conditions (Celenza et al. 1996; D’Amato et al. 2013; Ferrari et al. 2012; Michelozzi et al. 2007; Qiu et al. 2013; Spence et al. 1993; Tseng et al. 2013). In a study of 25 million ambulatory visits by COPD patients, temperature, wind speed, air pressure at sea level, and humidity had a significant influence in 1–2% of the ambulatory visits (Ferrari et al. 2012).

The weather is for a large part determined by barometric pressure (Barry and Chorley 2009). When the atmospheric pressure is decreased, the oxygen saturation is decreased as well (Horiuchi et al. 2018). Even moderate altitude has been found to be associated with a small decrease in oxygen saturation. Goldberg et al. found a mean oxygen saturation of 98.1% in a population of healthy young adults at 750 m altitude, compared to 98.5% in a comparable population at 43 m altitude (Goldberg et al. 2012).

A study with recreational climbers indicated significant changes in pulse oximetry, peak flow, and heart rate when reaching a height of about 1000 m (Napoli et al. 2009). However, no shortness of breath, also called dyspnea, could be observed during a state of hypobaric hypoxia at rest in healthy individuals (Nakano et al. 2015).

The aim of our study was to determine the influence of daily atmospheric pressure changes on oxygen saturation and shortness of breath in a general population at sea level.

## Materials and methods

### Population

**Fig. 1:**
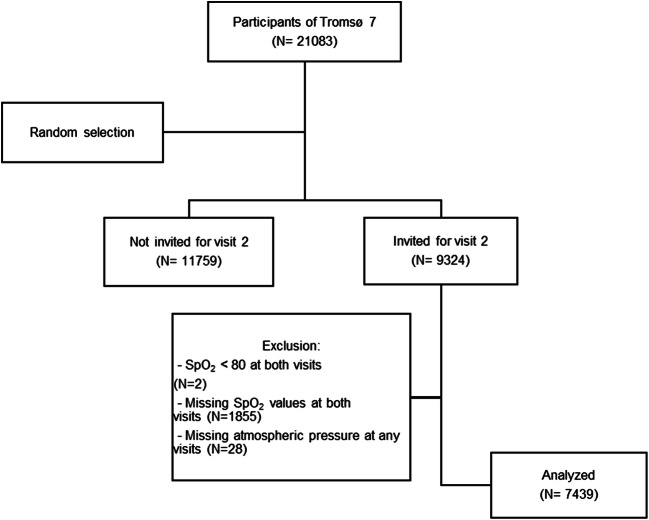
Flowchart indicating selection of study participants

Participants of the 7th survey of the Tromsø study were included. Tromsø 7 took place between March 2015 and October 2016 in Tromsø, a city at sea level in the northern part of Norway. All inhabitants in Tromsø aged 40 years or more were invited to the survey and 65% attended. A total of 21,083 participants were thus included. After a random selection procedure, with increased coverage of those age 60 years or more, 9324 participants were invited to a second visit with extended examinations. Included in this study were those with valid pulse oximetry at both visits measured on days with barometric pressure data available (Fig. 1). The Regional Committee for Medical and Health Research Ethics in North Norway approved Tromsø 7 survey. All participants gave written informed consent.

### Questionnaires

The participants answered questions on self-reported diseases such as chronic respiratory and cardiovascular diseases, smoking habit, chronic cough, and shortness of breath, using the modified Medical Research Council (mMRC) questionnaire. At visit 2, there was a computerized questionnaire at the spirometry station, with questions on symptoms of airway infection the previous week and on change in shortness of breath—How is your breathing today compared to normal: less short of breath, as normal, or more short of breath.

### Measurements

The atmospheric pressure was measured, at Tromsø Airport, Langnes, in hPa for every day during the Tromsø study by the Norwegian Meteorological Institute, and values corresponding to dates of visit 1 or 2 were used. The oxygen saturation was measured with a digital handheld pulse oximeter (Onyx II, model 9559; Nonin Medical, Inc.; Plymouth, MN, USA) at visit 1 and 2. The participant had to rest 15 min before the examination. The best of three measurements was used. Decreased oxygen saturation was defined as SpO_2_ ≤ 95% (Vold et al. 2012), and values below 80% were regarded as invalid. To determine the lung function of the participants, spirometry was performed using SensorMedics Vmax 20c Encore system (Viasys Healthcare Respiratory Technologies, Yorba Linda, CA, USA). The American Thoracic Society/European Respiratory Society criteria were followed (Miller et al. 2005). Calibration was performed every morning. Three trained technicians conducted the spirometry alternately. The participants were sitting and used a nose clip during the measurement. At least three exhalations were required. The difference between the best and second-best FEV1 was used and FVC was not to exceed 5% or 150 mL. Current drug therapy was not interrupted before the test. Reference values from GLI 2012 were used (Langhammer et al. 2016).

### Statistical analysis

Patient characteristics were compared by using independent t-test and Chi-square test for trend. Participants with and without decreased lung function, defined as FEV1/FVC < LLN or FEV1 < LLN, were compared. The barometric pressures were divided into quartiles, and oxygen saturation was dichotomized into normal and decreased values (SpO_2_ < 96%). A linear regression was performed to determine the association between the change in atmospheric pressure between visit 1 and visit 2 and the corresponding change in oxygen saturation. The association between change in atmospheric pressure between visit 1 and visit 2 and increased shortness of breath at visit 2 (as outcome) was analysed with logistic regression. Multivariate models were applied with possible confounders added to atmospheric pressure: self-reported heart attack, COPD, asthma, current smoking, symptoms of airway infection the week before visit 2, chronic shortness of breath (mMRC≥2), and significantly reduced lung function as measured by spirometry. Also age and sex were included in the models. In all analyses, IBM SPSS version 24 was used and a *p* value below 0.05 was considered statistically significant.

## Results

Among the 7439 participants included, 4065 were women (54.6%) and 3374 were men (45.4%). The mean number of days between the first and the second visit was 50 (SD 34.8). According to spirometry, 995 subjects had reduced lung function. The percentages with self-reported diseases such as asthma, COPD, and smoking were higher in participants with reduced lung function (Table 1).

**Table 1:** Participant characteristics

	Total *N* = 7439	Reduced lung function *N* = 995	No reduced lung function *N* = 6271	*p* value
Age (mean)	63.22 (40–84)	63.73 (40–84)	63.02 (40–84)	0.048^a^
Sex				0.505^b^
Women	4065 (54.6%)	532 (13.4%)	3424 (86.6%)	
Men	3374 (45.4%)	463 (14.0%)	2847 (86.0%)	
SpO_2_ (mean)				
Visit 1	97.8 (87–100)	97.33 (87–100)	97.89 (89–100)	< 0.001a
Visit 2	97.62 (84–100)	97.22 (89–100)	97.69 (90–100)	< 0.001a
Increased dyspnea at visit 2				< 0.001^b^
Yes	847 (11.4%)	185 (22.9%)	624 (77.1%)	
No	6592 (88.6%)	810 (12.5%)	5647 (87.5%)	
Smoking				< 0.001^c^
Yes	901 (12.1%)	258 (29.4%)	619 (70.6%)	
Previously	3603 (48.4%)	522 (14.9%)	2988 (85.1%)	
Never	2857 (38.4%)	208 (7.4%)	2596 (92.6%)	
mMRC ≥ 2				< 0.001^b^
Yes	376 (5.1%)	121 (35.0%)	225 (65.0%)	
No	7047 (94.7%)	873 (12.6%)	6031 (87.4%)	
Self-reported disease:				
Asthma				< 0.001^b^
Yes	823 (11.1%)	241 (30.4%)	551 (69.6%)	
No	6353 (85.4%)	710 (11.4%)	5506 (88.6%)	
COPD				< 0.001^b^
Yes	313 (4.2%)	167 (56.6%)	128 (43.4%)	
No	6817 (91.6%)	770 (11.5%)	5901 (88.5%)	
Symptoms of airway infection the previous 7 days at visit 2:				< 0.001^b^
Yes	1077 (14.5%)	182 (17.4%)	865 (82.6%)	
No	6340 (85.2%)	809 (13.1%)	5388 (86.9%)	
Heart attack				0.003^b^
Yes	365 (4.9%)	67 (18.9%)	287 (81.1%)	
No	6788 (91.2%)	885 (13.3%)	5747 (86.7%)	

*p values* according to ^a^independent t-test, ^b^χ2 and ^c^ χ2 for trend, as indicated

### Atmospheric pressure

**Fig. 2:**
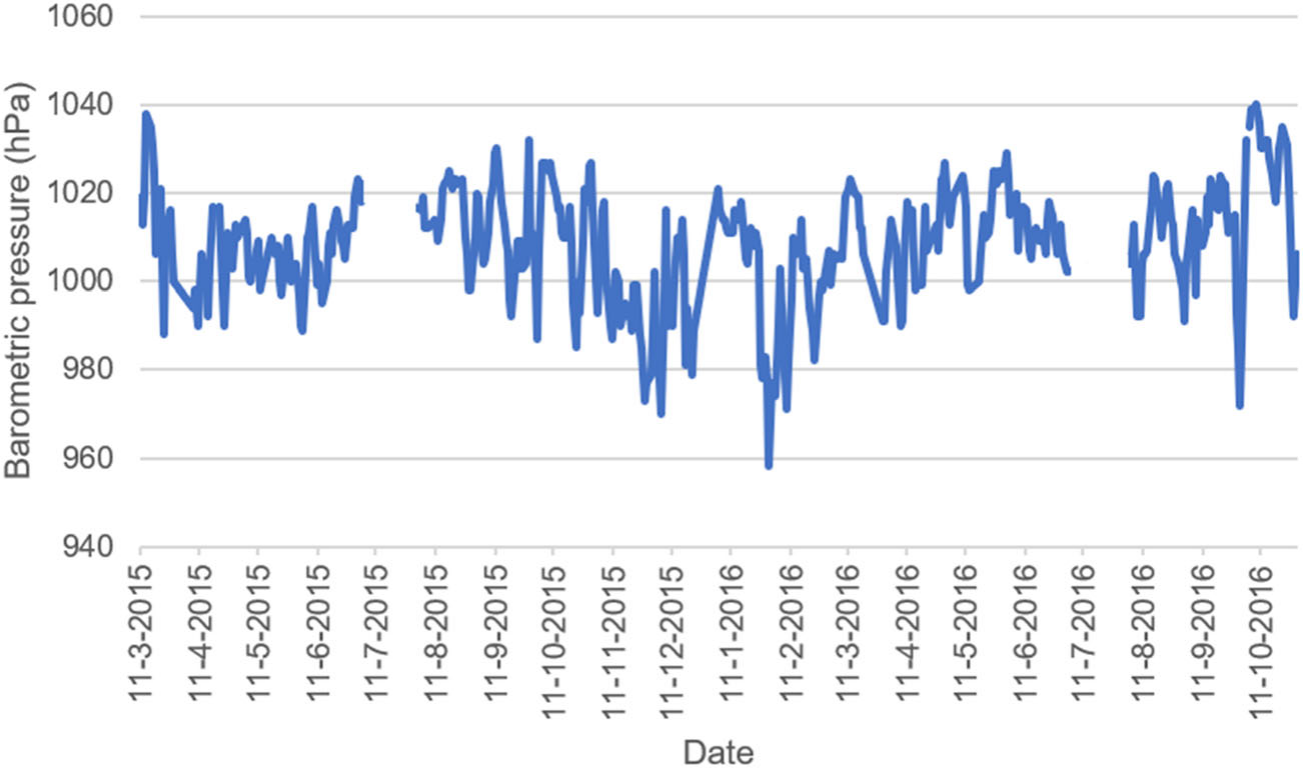
Barometric pressure day by day during Tromsø 7. Missing values are due to a break of the study in the months of July

The atmospheric pressure ranged from 958 hPa to 1039 hPa (Fig. 2). The mean was 1007.3 at first visit and 1007.9 at second visit.

### Prevalence of decreased oxygen saturation (SpO_2_ < 96%)

**Fig. 3:**
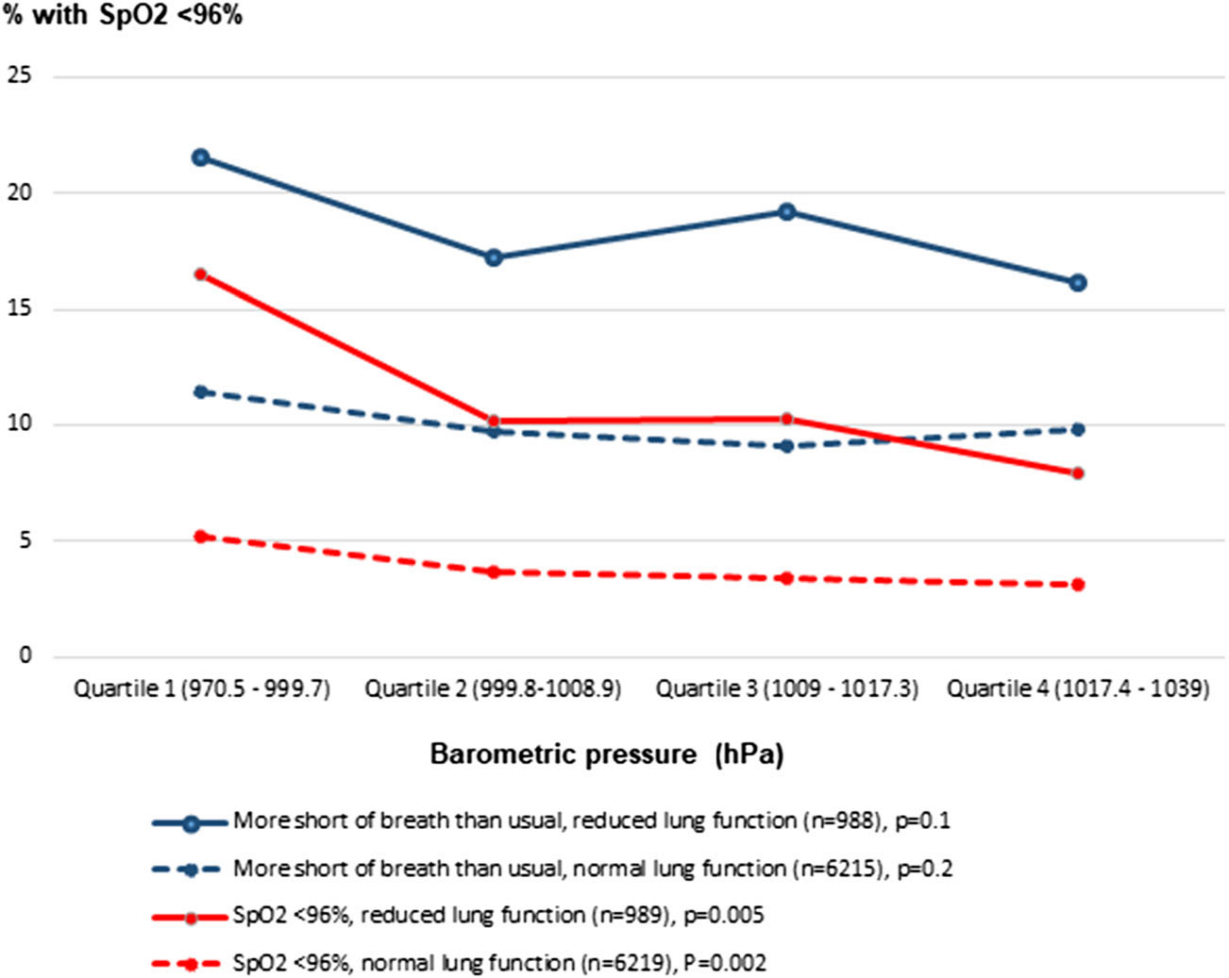
Association between barometric pressure (hPa, quartiles) and the frequency of SpO_2_ < 96% and increased shortness of breath at visit 2 in participants with normal and reduced lung function. Chi-square test for trend has been applied

The prevalence of SpO_2_ < 96% was 5.0%, and significantly higher in those with reduced lung function, 11.1% than in those with normal lung function, 3.8% (*p* < 0.001) (data not shown in table). The prevalence decreased with increasing quartile of barometric pressure. This is shown in Fig. 3 for visit 2, where this association was statistically significant for both participants with normal lung and reduced lung function, *p* = 0.002 and *p* = 0.005, respectively. Similar results were found at visit 1, but with nonsignificant association in the group with reduced lung function.

### Increased shortness of breath

Among all participants, 11.4% reported to be more short of breath than usual at visit 2. There was a tendency of decreasing frequency of increased dyspnea with increasing barometric pressure (Fig. 3), but the association was not statistically significant, neither among participants with normal lung function (*p* = 0.2) nor among those with reduced lung function (*p* = 0.1).

### Change in oxygen saturation

**Fig. 4:**
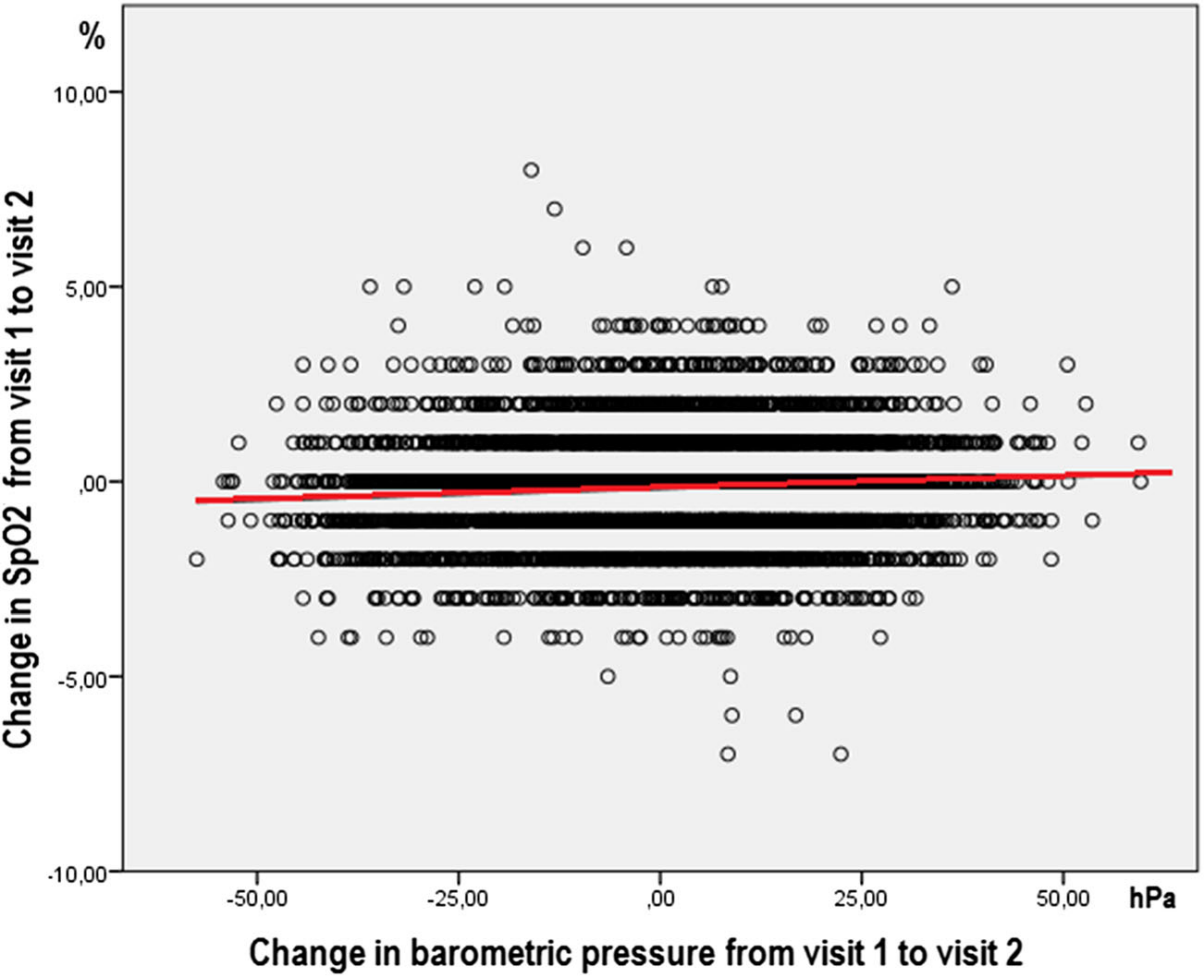
The association between change in barometric pressure (hPa) and change in SpO2 (%) between visit 1 and 2, β = 0.006

The SpO_2_ value was unchanged between visit 1 and visit 2 in 2845 participants (38.2%), 1% increase or decrease was found in 42.1%, a 2% change in 14.8%, and a change of 3% or more in 4.8%. Linear regression showed a significant association between the change in barometric pressure and change in SpO_2_ with β = 0.006 (*p* < 0.001; 95% CI = 0.004–0.008). This means that a reduction of 1 hPa in barometric pressure results in a reduction of 0.006 in oxygen saturation (Fig. 4), and that a reduction of 166.7 hPa was needed to get a 1% reduction in SpO_2_.

For participants with reduced lung function, the association was also significant β = 0.007 (*p* = 0.006; 95% CI = 0.002–0.012).

When adjusted for possible confounders in multivariable analysis, change in atmospheric pressure remained a significant predictor of change in SpO_2_ (Table 2).

**Table 2:** Multivariable analysis for change in SpO_2_

	β unadjusted (95% CI)	*p* value	β adjusted (95% CI)	*p* value
Change atmospheric pressure	0.006 (0.004–0.008)	< 0.001	0.006 (0.005–0.008)	< 0.001
Age	0.002 (0.000–0.005)	0.1	0.002 (0.000–0.005)	0.09
Sex				
Woman	Reference			
Men	− 0.022 (− 0.078–0.035)	0.4	− 0.012 (− 0.071–0.047)	0.7
Smoking				
No currently	Reference			
Yes	0.083 (− 0.003–0.169)	0.06	0.067 (− 0.024–0.158)	0.2
Asthma				
No	Reference			
Yes	0.049 (− 0.040–0.139)	0.3	0.026 (− 0.072–0.124)	0.6
COPD				
No	Reference			
Yes	0.125 (− 0.015–0.264)	0.08	− 0.002 (− 0.126–0.158)	1.0
Heart attack				
No	Reference			
Yes	0.017 (− 0.113–0.147)	0.8	0.014 (− 0.125–0.153)	0.8
mMRC ≥ 2				
No	Reference			
Yes	0.211 (0.083–0.339)	0.001	0.183 (0.039–0.327)	0.01
Reduced lung function				
No	Reference			
Yes	0.084 (0.001–0.166)	0.05	0.049 (− 0.042–0.140)	0.3
Airway infection visit 2				
No	Reference			
Yes	− 0.013 (− 0.093–0.067)	0.7	− 0.030 (− 0.114–0.054)	0.5

### Increased shortness of breath and change in barometric pressure

In the multivariable logistic regression with increased shortness of breath at visit 2 as outcome, change in barometric pressure was not a significant predictor. The strongest predictors were recent airway infection, mMRC, and smoking currently. Age, asthma, and reduced lung function were also significant predictors (Table 3).

**Table 3:** Multivariable analysis for increased dyspnea

	OR (95% CI)	*p* value	OR adjusted (95% CI)	*p* value
Change atmospheric pressure	1.000 (0.996–1.005)	0.862	1.001 (0.996–1.005)	0.7
Age	0.990 (0.983–0.997)	0.004	0.988 (0.980–0.996)	0.002
Sex				
Woman	Reference			
Men	1.054 (0.913–1.217)	0.5	1.083 (0.917–1.279)	0.3
Smoking currently				
No	Reference			
Yes	2.306 (1.924–2.763)	< 0.001	1.887 (1.525–2.334)	< 0.001
Asthma				
No	Reference			
Yes	1.964 (1.618–2.384)	< 0.001	1.526 (1.198–1.944)	0.001
COPD				
No	Reference			
Yes	2.271 (1.715–3.007)	< 0.001	1.248 (0.874–1.782)	0.2
Heart attack				
No	Reference			
Yes	1.019 (0.733–1.418)	0.9	0.859 (0.578–1.275)	0.5
mMRC ≥ 2				
No	Reference			
Yes	3.819 (3.027–4.818)	< 0.001	3.643 (2.713–4.890)	< 0.001
Reduced lung function				
No	Reference			
Yes	2.067 (1.727–2.474)	< 0.001	0.118 (1.022–1.604)	0.03
Airway infection visit 2				
No	Reference			
Yes	6.860 (5.865–8.024)	< 0.001	6.858 (5.777–8.141)	< 0.001

## Discussion

Atmospheric pressure at sea level was significantly associated with oxygen saturation. This was the case in participants with reduced lung function as well as in those with normal lung function, as measured by spirometry. A reduction in atmospheric pressure was not associated with increased dyspnea.

To get a 1% reduction in SpO_2,_ a drop in barometric pressure of 166.67 hPa was needed. A similar drop can be attained when climbing from sea level to an altitude of 1400 m, as, normally, barometric pressure decreases by about 12 hPa per 100 m in the first 1000 m, using the formula for pressure and height (NASA 1976; Quick 2004). Our results correspond to the difference in oxygen saturation of 0.42% when measurements at 725 m and 43 m were compared (Goldberg et al. 2012). Greater effect of change in altitude has been found in individuals ascending to higher altitudes. One study of 19 healthy adults showed SpO_2_ values at 2305 m of 93.8% dropping to 90.2% at 3000 m (Horiuchi et al. 2018). In another study, ascending towards an altitude of 2100 m (from 490 m to 2590 m) led to a decrease of 4% in SpO_2,_ measured in healthy men in the evening (Stadelmann et al. 2015). The barometric pressure dropped from 959 hPa (719 Torr) to 749 hPa (562 Torr) which means that a change of 52,5 hPa was needed for a change of 1% in SpO_2_. The starting point before change in altitude may play a role, and at the same natural changes in barometric pressure might have a greater impact on oxygen saturation at high altitude than at sea level. A particularly large drop in oxygen saturation has been found in COPD patients, from a mean of 96% at sea level to 87% at 2438 m, with further decrease during exercise (Christensen et al. 2000).

The change in barometric pressure in our study was not associated with shortness of breath, also not in participants with decreased lung function. This has previously been found in healthy adults (Nakano et al. 2015). There are considerable effects of altitude in patients with cardiac or respiratory disease. Asthma patients can develop moderate loss of asthma control, increased airway obstruction and neutrophilic airway inflammation (Seys et al. 2013). Development of severe hypoxia was described by Christensen and co-workers (Christensen et al. 2000), but the association with increased dyspnea was not studied. We still do not know whether shortness of breath can be induced at high altitude in patients with cardiac or respiratory diseases.

### Strengths and limitations

The large study population is an important strength, also the routine collection of barometric pressures carried out independent of the Tromsø study. There are some limitations. A limitation is that not all kinds of weather circumstances were included, only the atmospheric pressure. It is known from studies that temperature, humidity, etc. have an influence on respiratory symptoms (Celenza et al. 1996; D’Amato et al. 2013; Ferrari et al. 2012; Michelozzi et al. 2007; Qiu et al. 2013; Spence et al. 1993; Tseng et al. 2013). Questionnaires were used to collect information on shortness of breath and possible confounders, like smoking and self-reported diseases, which means that recall bias could be a problem. Another limitation was the attendance rate of Tromsø 7. Although higher than in most population surveys, under representation of those too sick to attend could lead to bias. It is also a limitation that, concerning external validity, the sample consisted only of Norwegians. The close to polar climate in Tromsø may have affected the influence of other weather characteristics than the barometric pressure.

## Conclusion

Atmospheric pressure was strongly associated with oxygen saturation at sea level, but the association was weaker than previously found at high altitudes. Change in atmospheric pressure was not a predictor for reporting increased dyspnea, also not in subjects with reduced lung function. More research is needed to determine the effect of atmospheric pressure on increased dyspnea.
